# Ovarian stimulation for IVF and risk of primary breast cancer in *BRCA1/2* mutation carriers

**DOI:** 10.1038/s41416-018-0139-1

**Published:** 2018-06-25

**Authors:** Inge A. P. Derks-Smeets, Lieske H. Schrijver, Christine E. M. de Die-Smulders, Vivianne C. G. Tjan-Heijnen, Ron J. T. van Golde, Luc J. Smits, Beppy Caanen, Christi J. van Asperen, Margreet Ausems, Margriet Collée, Klaartje van Engelen, C. Marleen Kets, Lizet van der Kolk, Jan C. Oosterwijk, Theo A. M. van Os, HEBON, Matti A. Rookus, Flora E. van Leeuwen, Encarna B. Gómez García

**Affiliations:** 1 Department of Clinical Genetics, Maastricht University Medical Centre+, P.O. Box 5800, 6202 AZ Maastricht, The Netherlands; 2 GROW – School for Oncology and Developmental Biology, Maastricht University, P.O. Box 616, 6200 MD Maastricht, The Netherlands; 3 Department of Epidemiology, Netherlands Cancer Institute, Plesmanlaan 121, 1066 CX Amsterdam, Netherlands; 4 Department of Internal Medicine, Division of Medical Oncology, Maastricht University Medical Centre+, P.O. Box 5800, 6202 AZ Maastricht, The Netherlands; 5 Department of Obstetrics and Gynaecology, Maastricht University Medical Centre+, P.O. Box 5800, 6202 AZ Maastricht, The Netherlands; 6 Department of Epidemiology, Maastricht University, P.O. Box 616, 6200 MD Maastricht, The Netherlands; 7 Department of Clinical Genetics, Leiden University Medical Centre Leiden, P.O. Box 9600, 2300 RC Leiden, The Netherlands; 8 Department of Genetics, University Medical Centre Utrecht, P.O. 85500, 3508 GA Utrecht, The Netherlands; 9 Department of Clinical Genetics, Erasmus University Medical Center, P.O. Box 2040, 3000 CA Rotterdam, The Netherlands; 10 Department of Clinical Genetics, VU University Medical Centre, P.O. Box 7057, 1007 MB Amsterdam, The Netherlands; 11 Department of Human Genetics, Radboud University Medical Centre, P.O. Box 9101, 6500 HB Nijmegen, The Netherlands; 12 Family Cancer Clinic, Netherlands Cancer Institute, Plesmanlaan 121, 1066 CX Amsterdam, The Netherlands; 13 Department of Genetics, University of Groningen, University Medical Center Groningen, P.O. Box 30.001, 9700 RB Groningen, The Netherlands; 14 Department of Clinical Genetics, Academic Medical Centre, P.O. Box 22700, 1100 DE Amsterdam, The Netherlands

**Keywords:** Risk factors, Cancer epidemiology, Breast cancer, Epidemiology, Breast cancer

## Abstract

**Background:**

The effect of in vitro fertilisation (IVF) on breast cancer risk for *BRCA1/2* mutation carriers is rarely examined. As carriers may increasingly undergo IVF as part of preimplantation genetic diagnosis (PGD), we examined the impact of ovarian stimulation for IVF on breast cancer risk in *BRCA1/2* mutation carriers.

**Methods:**

The study population consisted of 1550 *BRCA1* and 964 *BRCA2* mutation carriers, derived from the nationwide HEBON study and the nationwide PGD registry. Questionnaires, clinical records and linkages with the Netherlands Cancer Registry were used to collect data on IVF exposure, risk-reducing surgeries and cancer diagnosis, respectively. Time-dependent Cox regression analyses were conducted, stratified for birth cohort and adjusted for subfertility.

**Results:**

Of the 2514 BRCA1/2 mutation carriers, 3% (*n* = 76) were exposed to ovarian stimulation for IVF. In total, 938 *BRCA1/2* mutation carriers (37.3%) were diagnosed with breast cancer. IVF exposure was not associated with risk of breast cancer (HR: 0.79, 95% CI: 0.46–1.36). Similar results were found for the subgroups of subfertile women (*n* = 232; HR: 0.73, 95% CI: 0.39–1.37) and *BRCA1* mutation carriers (HR: 1.12, 95% CI: 0.60–2.09). In addition, age at and recency of first IVF treatment were not associated with breast cancer risk.

**Conclusion:**

No evidence was found for an association between ovarian stimulation for IVF and breast cancer risk in *BRCA1/2* mutation carriers.

## Introduction

Women with a mutation in the *BRCA1* or *BRCA2* gene have an increased risk of breast, ovarian and other types of cancer.^1,2^ Both exogenous and endogenous oestrogens and progestogens have been shown to affect breast cancer risk in both the general population and mutation carriers.^3–7^ In vitro fertilisation (IVF) might influence breast cancer risk, as the use of a long agonist protocol for ovarian stimulation for IVF results in a period of decreased levels of oestrogen and progesterone due to downregulation of the natural hormonal cycle,^8^ followed by a temporary hyperoestrogenic state.^9^ Hypothetically, this hyperoestrogenic environment might be carcinogenic, as are prolonged cyclic changes in oestrogen and progesterone levels. For *BRCA1/2* mutation carriers not only infertility, but also oncologic treatment strategies affecting fertility and/or risk-reducing salpingo-oophorectomy direct female mutation carriers to ovarian stimulation for IVF for fertility preservation.^10,11^ In addition, during the past decade the request for preimplantation genetic diagnosis (PGD) has increased rapidly for *BRCA1/2* mutation carriers, i.e., genetic testing of embryos for the presence of a known familial mutation before transfer in order to establish a pregnancy of a foetus without the genetic condition.^12–14^

In the general population, recent meta-analyses did not report an association between exposure to ovarian stimulation for IVF and breast cancer risk.^15,16^ A large recent cohort study in over 19,000 Dutch women did also not show an adverse effect of IVF on breast cancer risk.^17^

For women with a *BRCA1/2* mutation the association between exposure to ovarian stimulation for IVF and breast cancer risk has hardly been studied. The only case–control study conducted did not find an elevated risk of breast cancer associated with IVF exposure.^18^ As women with *BRCA1/2* mutations have a high a priori absolute risk of breast cancer development already at early, reproductive ages (cumulative risks of 20% by age 38 and 43 apply for *BRCA1* and *BRCA2*, respectively,^1^ even a small adverse effect of ovarian stimulation for IVF could have substantial impact. This is all the more important given the high prevalence of heterozygous *BRCA1/2* germline mutations (i.e., 1:400 up to 1:200 persons in some general populations^19^ and 1:40 in certain ethnic groups.^20^ The aim of the current study is to evaluate whether exposure to ovarian stimulation for IVF increases the risk of primary breast cancer in *BRCA1* and *BRCA2* mutation carriers.

## Patients and methods

Women were eligible if they were 18 years or older and had been tested positive for a pathogenic mutation in either the *BRCA1* or *BRCA2* gene. Women born before 1940 were excluded because they had their reproductive years before IVF was available.

Our study population was derived from two sources: the Dutch HEBON study (Hereditary Breast and Ovarian cancer study, the Netherlands) and the national PGD registry.

The HEBON study (initiated in 1999) is an ongoing nationwide retrospective cohort study among members of *BRCA1/2* mutation families with prospective follow-up.^21^ Participants were invited into the study after they became aware of their mutation carrier status through a clinical genetic test. Current analyses were restricted to participants that entered the HEBON cohort between 2010 and 2013, since only the most recent HEBON baseline questionnaire included questions concerning exposure to fertility treatments; response to the most recent HEBON questionnaire was 57%. In the Netherlands, PGD for *BRCA1/2* mutations has been offered since 2008 in Maastricht University Medical Centre, in collaboration with IVF transport clinics in University Medical Centres of Utrecht and Groningen, and the Academic Medical Centre Amsterdam. All women who undergo PGD are registered in the PGD registry. Some women were eligible through the HEBON study and had also been registered in the PGD registry.

The HEBON study was approved by the medical ethics committees of all participating Dutch University Medical Centres or Family Cancer Clinics. All HEBON participants gave their informed consent for linkage with the nationwide cancer and pathology registries. The PGD programme has been approved by the Institutional Review Board of Maastricht University Medical Centre. All patients undergoing PGD gave their written informed consent for this treatment and use of their medical data for scientific research.

### Data collection

For *BRCA1/2* mutation carriers included through the HEBON study, data on exposure to IVF and other fertility treatments (i.e., ovulation induction and intra-uterine insemination), prophylactic surgeries and confounders were collected with the HEBON baseline questionnaire. In case of female subfertility (defined as the inability to establish a clinical pregnancy within 12 or more months of regular, unprotected sexual intercourse with the intention to conceive), women reported the type(s) of fertility treatment(s) they underwent and their age at first and last treatment. Information on cancer history was self-reported but also obtained by linkage with both the Dutch national Pathology Database (PALGA) and the Netherlands Cancer Registry (NCR) for the period after 1988. For the period when these registries were not yet available (≤1988), cancer diagnoses were solely self-reported. For the women included through the PGD registry, data on exposure (type of fertility treatment(s) and age at treatments), potential confounders, prophylactic surgeries and cancer history were retrieved from medical files.

For women eligible through the HEBON study and also registered in the PGD registry, the data from medical files and the HEBON questionnaire were combined. No conflicting data were found.

### Statistical analysis

The association between IVF exposure and risk of breast cancer was analysed using a time-dependent Cox proportional hazards regression model with age as the timescale. Main analyses were stratified for birth cohort (based on tertiles of number of carriers per category: 1940–1957, 1958–1968 and 1969–1993) and adjusted for subfertility (no, yes or missing). Observation time started at birth and ended at age at diagnosis of first invasive breast cancer (event of interest), other invasive cancer diagnosis (excl. basal cell carcinoma) or bilateral prophylactic mastectomy (BPM), whichever occurred first. In case these events did not take place before baseline questionnaire or last PGD contact, observation time ended at the age of baseline questionnaire completion or last PGD contact, whichever was last.

The exposed group, in terms of person-time, consisted of observation time of *BRCA1/2* mutation carriers who had undergone ovarian stimulation for IVF before censoring. Women were considered as exposed to IVF from the first IVF treatment onward. The unexposed (comparison) person-time consisted of observation time of *BRCA1/2* mutation carriers who had not (yet) been exposed to ovarian stimulation for IVF before censoring. Cutoffs of categories in variables related to IVF characteristics are based on number of cases available in the exposed group.

Potential confounders, besides subfertility and birth cohort, were exposure to other fertility treatments (clomid and/or intra-uterine insemination (in which hormonal treatment may be included)), (time-dependent: ever/never), use of oral contraceptives (time-dependent: ever/never), parity (time-dependent: nulliparous/parous) and age at first childbirth (time-dependent: nulliparous/<25 years/25–29 years/30+ years). None of these changed the hazard ratio (HR) for IVF treatment by more than 10% and therefore they were not included. The uptake of an RRSO could only be tested as a potential confounder in an unbiased manner by comparing univariate and multivariate analyses from analyses between IVF uptake and breast cancer risk with restricted follow-up time until age at DNA test, since the uptake of RRSO mainly depends on the outcome of the DNA test.^22^ As univariate and multivariate HRs were similar (<10% change), RRSO uptake was not considered to be a confounder.

*BRCA* mutation-specific analyses were only conducted in the subgroup of *BRCA1* mutation carriers since power in *BRCA2* carriers was too limited. An additional sensitivity analysis restricted to subfertile women was conducted to further assess any association in this stratum.

All statistical analyses were performed using STATA version 13 (StataCorp, College Station, TX).

## Results

### Patient characteristics

**Fig. 1:**
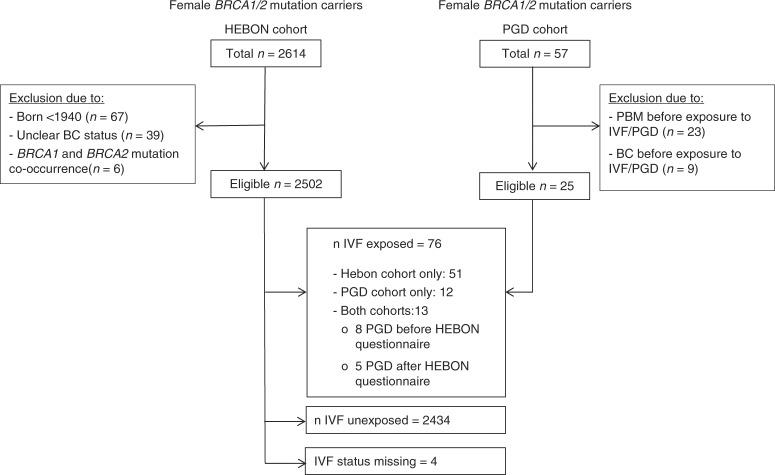
Composition of study groups

The final group eligible for analysis comprised 2514 *BRCA1/2* mutation carriers (1550 *BRCA1* and 964 *BRCA2*). Of these, 2502 (99.5%) women participated in the HEBON study. The PGD group included a total of 25 eligible women, including 13 responders to the HEBON study (Fig. 1).

In total, 938 of 2514 women (37.3%) were diagnosed with breast cancer, 630/1550 *BRCA1* (40.7%) and 308/964 *BRCA2* mutation carriers (32.0%, Table 1). The mean age at breast cancer diagnosis was 40.1 years for *BRCA1* and 44.4 years for *BRCA2* mutation carriers. Of the remaining participants, 163 (10.3%) were censored at the age of another cancer diagnosis, 423 (26.8%) at the age of BPM and 990 women (26.8%) at the age of the questionnaire or last PGD contact. Their mean age at censoring was 43.7 years (*BRCA1* 43.1 years and *BRCA2* 44.6 years). Those women only identified through the PGD registry were on average younger at censoring compared to the women included through the HEBON study (33.0 years versus 43.0 years). Of all mutation carriers, women unaffected with breast cancer were slightly more often born in more recent birth years (1969–1993: 43.4% mutation carriers unaffected with breast cancer versus 22.3% affected with breast cancer).

**Table 1:** Cohort characteristics of *BRCA1/2* mutations carriers

	BRCA1 (*n* = 1550)		BRCA2 (*n* = 964)		BRCA1/2 combined (*n* = 2514)	
	BC+^a^	BC−^a^	BC+^a^	BC−^a^	BC+^a^	BC−^a^
*n* (%)	630 (40.7)	920 (59.3)	308 (31.9)	656 (68.1)	938 (37.3)	1576 (62.7)
Data source, *n* (%)						
HEBON	629 (99.8)	913 (99.2)	308 (100)	639 (97.4)	937 (99.9)	1552 (98.5)
HEBON + PGD:						
PGD < HEBON	1 (0.2)	2 (0.2)	0 (0.0)	5 (0.8)	1 (0.1)	7 (0.4)
PGD > HEBON	0 (0.0)	1 (0.1)	0 (0.0)	4 (0.6)	0 (0.0)	5 (0.3)
PGD only	0 (0.0)	4 (0.4)	0 (0.0)	8 (1.2)	0 (0.0)	12 (0.8)
Age at censoring (years) (%)						
Mean age at censoring (SD)	40.1 (8.5)	43.1 (11.6)	44.4 (8.9)	44.6 (12.0)	41.5 (8.9)	43.7 (11.8)
<35	176 (27.9)	247 (26.9)	36 (11.7)	149 (22.7)	212 (22.6)	396 (25.1)
35–44	276 (43.8)	259 (28.2)	132 (42.9)	190 (29.0)	408 (43.5)	449 (28.5)
45–64	175 (27.8)	384 (41.7)	139 (45.1)	276 (42.1)	314 (33.5)	660 (41.9)
65+	3 (0.5)	30 (3.3)	1 (0.3)	41 (6.3)	4 (0.4)	71 (4.5)
Censoring reason						
Baseline Q/PGD contact	0 (0.0)	561 (61.0)	0 (0.0)	429 (65.4)	0 (0.0)	990 (62.8)
Breast cancer	630 (100)	0 (0.0)	308 (100)	0 (0.0)	938 (100)	0 (0.0)
Other cancer	0 (0.0)	90 (9.8)	0 (0.0)	73 (11.1)	0 (0.0)	163 (10.3)
BPM	0 (0.0)	269 (29.2)	0 (0.0)	154 (23.5)	0 (0.0)	423 (26.8)
Birth year (%)						
1940–1957	244 (38.7)	216 (23.5)	153 (49.7)	191 (29.1)	397 (42.3)	407 (25.8)
1958–1968	231 (36.7)	296 (32.2)	101 (32.8)	189 (28.8)	332 (35.4)	485 (30.8)
1969–1993	155 (24.6)	408 (44.4)	54 (17.5)	276 (42.1)	209 (22.3)	684 (43.4)
IVF (%)						
No	616 (97.8)	889 (96.6)	305 (99.0)	624 (95.1)	921 (98.2)	1513 (96.0)
Yes	12 (1.9)	29 (3.2)	3 (1.0)	32 (4.9)	15 (1.6)	61 (3.9)
Missing	2 (0.3)	2 (0.2)	0 (0.0)	0 (0.0)	2 (0.2)	2 (0.1)

*BC* breast cancer, *SD* standard deviation, *Q* questionnaire, *IVF* in vitro fertilisation, *PGD* preimplantation genetic diagnosis.

^a^Distribution of variables at the time of censoring

In total, 3% (*n* = 76) of all *BRCA1/2* mutation carriers included were exposed to ovarian stimulation for IVF; 41 *BRCA1* and 35 *BRCA2* mutation carriers. For 51 mutation carriers, IVF exposure was identified through the HEBON study and for 25 through the PGD registry. Due to the nature of the IVF exposure question in the HEBON study questionnaire (subfertile women only), data on IVF exposure of eight mutation carriers were collected through the PGD registry while they were exposed before date of HEBON questionnaire. Mutation carriers were on average 31.7 years at first IVF treatment. At censoring, their first IVF treatment was on average 6.5 years ago (Table 2). The year of first treatment varied between 1989 and 2015, while mutation carriers included through the PGD registry received their first IVF treatment in more recent years (HEBON: 1989–2013, median 2003 and PGD: 2010–2015, median 2013). Mutation carriers exposed to IVF were more often subfertile (64.5% versus 7.4%) and nulliparous (36.8% versus 19.3%) compared to unexposed mutation carriers.

**Table 2:** IVF exposure and breast cancer risk in *BRCA1/2* mutation carriers

	BC+^a^	BC−^a^	HR (95% CI)^b^
*n* = 2514	938 (37.3)	1576 (62.7)	
IVF treatment			
No	921 (98.2)	1513 (96.0)	1.00
Yes	15 (1.6)	61 (3.9)	0.79 (0.46–1.36)
Missing	2 (0.2)	2 (0.1)	
Year at first IVF (years)			
Median (min–max)	2000 (1988–2010)	2005 (1985–2015)	
Age at first IVF (%)			
Median (min–max, years)	30.9 (23–37)	31.9 (24–42)	
No IVF	921 (98.2)	1513 (96.0)	1.00
≤32 years	9 (1.0)	36 (2.3)	1.13 (0.57–2.2)
>32 years	6 (0.6)	25 (1.6)	0.54 (0.24–1.24)
Missing	2 (0.2)	2 (0.1)	
Time since first IVF treatment (%)			
No IVF	921 (98.2)	1513 (96.0)	1.00
<5 years ago started	7 (0.8)	34 (2.2)	0.83 (0.39–1.78)
≥5 years ago started	8 (0.9)	27 (1.7)	0.75 (0.36–1.56)
Missing	2 (0.2)	2 (0.1)	
Time since last IVF treatment (%)			
No IVF	921 (98.2)	1513 (96.0)	1.00
<2 years ago stopped	6 (0.6)	20 (1.3)	0.99 (0.43–2.25)
≥2 years ago stopped	7 (0.8)	40 (2.5)	0.60 (0.28–1.30)
Age of last treatment before censoring unknown	2 (0.2)	1 (0.1)	
Missing	2 (0.2)	2 (0.1)	

*BC* breast cancer, *HR* hazard ratio, *CI* confidence interval, *IVF* in vitro fertilisation.

^a^Distribution of variable at time of censoring.

^b^Instrinsically stratiefied on birth year (1940–1957, 1958–1968, 1969–1993); adjusted for subfertility (no, yes, missing)

### Breast cancer risk in relation to IVF

At the end of follow-up, 15 mutation carriers (12 *BRCA1* and 3 *BRCA2*) exposed to ovarian stimulation for IVF had developed breast cancer. Exposure to ovarian stimulation for IVF was not associated with the risk of breast cancer (HR: 0.79, 95% CI: 0.46–1.36, Table 2). Also, specific IVF characteristics, e.g., age at first IVF treatment was not associated with breast cancer risk (no IVF, HR: 1.0 (reference), ≤32 years HR: 1.13, 95% CI: 0.57–2.20, >32 years HR: 0.54, 95% CI: 0.24–1.24; Table 2).

Overall, results of additional analyses in subgroups of *BRCA1* mutation carriers and in subfertile women were similar compared to results based on the total cohort. Both in *BRCA1* mutation carriers (HR: 1.12, 95% CI: 0.60–2.09; Table 3) and in subfertile women (HR: 0.73, 95% CI: 0.39–1.37; Table 4), IVF exposure was not associated with breast cancer risk. However, in *BRCA1* mutation carriers a younger age at first IVF treatment seemed to be more strongly related with breast cancer risk than first IVF treatment at older ages (≤32 years HR: 1.91, 95% CI: 0.88–4.16, >32 years HR: 0.70, 95% CI: 0.28–1.76), but numbers were small.

**Table 3:** IVF exposure and breast cancer risk in *BRCA1* mutation carriers

	BC+^a^	BC−^a^	HR (95% CI)^b^
*n* = 1550	630 (40.7)	920 (59.4)	
IVF treatment			
No	616 (97.8)	889 (96.6)	1.00
Yes	12 (1.9)	29 (3.2)	1.12 (0.60–2.09)
Missing	2 (0.3)	2 (0.2)	
Age at first IVF (%)^**c**^			
Median (min–max, years)	31.1 (23–36)	32.3 (24–42)	
No IVF	616 (97.8)	889 (96.6)	1.00
≤32 years	7 (1.1)	14 (1.5)	1.91 (0.88–4.16)
>32 years	5 (0.8)	15 (1.6)	0.70 (0.28–1.76)
Missing	2 (0.3)	2 (0.2)	
Time since first IVF treatment (%)^**c**^			
No IVF	616 (97.8)	889 (96.6)	1.00
<5 years ago started	6 (1.0)	13 (1.4)	1.15 (0.49–2.66)
≥5 years ago started	6 (1.0)	16 (1.7)	1.10 (0.47–2.56)
Missing	2 (0.3)	2 (0.2)	
Time since last IVF treatment (%)^**c**^			
No IVF	616 (97.8)	889 (96.6)	1.00
<2 years ago stopped	5 (0.8)	7 (0.8)	1.49 (0.60–3.73)
≥2 years ago stopped	5 (0.8)	21 (2.3)	0.84 (0.33–2.10)
Age of last treatment before censoring unknown	2 (0.3)	1 (0.1)	
Missing	2 (0.3)	2 (0.2)	

*BC* breast cancer, *HR* hazard ratio, *CI* confidence interval, *IVF* in vitro fertilisation.

^a^Distribution of variable at time of censoring.

^b^Instrinsically stratiefied on birth year (1940–1957, 1958–1968, 1969–1993); adjusted for subfertility (no, yes, missing).

^c^Cutoffs of categories in variables related to IVF characteristics are based on number of cases available in the exposed group

**Table 4:** IVF exposure and breast cancer risk in subfertile *BRCA1/2* mutation carriers

	BC+^a^	BC−^a^	HR (95% CI)^b^
*n* = 232	93 (40.1)	139 (59.9)	
IVF treatment			
No	79 (85.0)	100 (71.9)	1.00
Yes	12 (12.9)	37 (26.6)	0.73 (0.39–1.37)
Missing	2 (2.2)	2 (1.4)	
Age at first IVF (%)^**c**^			
Median (min–max, years)	31.9 (24–37)	32.6 (24–41)	
No IVF	79 (85.0)	100 (71.9)	1.00
≤32 years	6 (6.5)	17 (12.2)	0.93 (0.40–2.17)
>32 years	6 (6.5)	20 (14.4)	0.60 (0.25–1.41)
Missing	2 (2.2)	2 (1.4)	
Time since first IVF treatment (%)^**c**^			
No IVF	79 (85.0)	100 (71.9)	1.00
<5 years ago started	6 (6.5)	11 (7.9)	0.94 (0.40–2.22)
≥5 years ago started	6 (6.5)	26 (18.7)	0.59 (0.25–1.40)
Missing	2 (2.2)	2 (1.4)	
Time since last IVF treatment (%)^**c**^			
No IVF	79 (85.0)	100 (71.9)	1.00
<2 years ago stopped	5 (5.4)	7 (5.0)	1.05 (0.41–2.65)
≥2 years ago stopped	6 (6.5)	29 (20.9)	0.55 (0.23–1.31)
Age of last treatment before censoring unknown	1 (1.1)	1 (0.7)	
Missing	2 (2.2)	2 (1.4)	

*BC* breast cancer, *HR* hazard ratio, *CI* confidence interval, *IVF* in vitro fertilisation.

^a^Distribution of variable at time of censoring.

^b^Instrinsically stratiefied on birth year (1940–1957, 1958–1968, 1969–1993).

^c^Cutoffs of categories in variables related to IVF characteristics are based on numbers of cases available in the exposed group

## Discussion

We showed that for *BRCA1* and *BRCA2* mutation carriers breast cancer risk was not increased after exposure to ovarian stimulation for IVF. This was also the case when assessing this risk in subgroups of subfertile women and *BRCA1* mutation carriers alone.

The association between exposure to ovarian stimulation for IVF and the incidence of breast cancer in *BRCA1/2* mutation carriers was examined in only one previous study. Kotsopoulos et al.^18^ studied the risk of breast cancer associated with infertility, fertility treatment and IVF treatment in a case–control design. They included 1380 women with a *BRCA1* or *BRCA2* mutation with a history of breast cancer (cases) and matched them to 1380 female *BRCA1* or *BRCA2* mutation carriers without a history of breast cancer (controls). No association between exposure to IVF and breast cancer risk was observed (multivariable odds ratio: 0.98, 95% CI: 0.39–2.45, based on nine cases who received IVF), while a non-significantly increased association was found between exposure to gonadotropin-containing fertility medication and breast cancer risk (multivariable odds ratio: 2.32, 95% CI: 0.91–5.95). Data regarding types of fertility treatment and medication used were self-reported in Kotsopoulos et al. and information concerning the latter was missing in 27% of the study subjects. Our data regarding type of fertility treatment was self-reported as well for the greater part of exposed women, and we did not collect information regarding the type of medication used. In the Netherlands, IVF is in the vast majority of cases performed using gonadotropins for ovarian stimulation. Gonadotropins are also used but to a lesser extent for ovulation induction, as a second choice for clomiphene citrate-containing drugs (e.g., clomid). In the study by Kotsopoulos et al., it was not described for which fertility treatments gonadotropin-containing medications were used, nor which types of medication were used for ovarian stimulation for IVF. Additionally, no information regarding the indication for fertility treatment was provided. As a consequence, it is difficult to interpret their apparent different findings between exposure to IVF treatment and exposure to gonadotropin-containing drugs. Only 9 and 15 cases and 11 and 61 controls were exposed to IVF in the study of Kotsopoulos et al. and our study, respectively. Thus, the power in the present study is larger but still too limited to exclude a true weak association.

The association between ovarian stimulation for IVF and the risk of breast cancer has been extensively studied in the non-*BRCA* population. In a recently published cohort study in over 19,000 Dutch women, the risk of breast cancer after exposure to ovarian stimulation for IVF was neither different from the risk of breast cancer in the general population (standardised incidence ratio (SIR): 1.01, 95% CI: 0.93–1.09) nor from the risk of breast cancer in a non-IVF subfertile comparison group (HR: 1.01, 95% CI: 0.86–1.19).^17^ A recent meta-analysis^16^ drew the same conclusion: in a cumulative cohort of over 1.5 million women, no association was found between ovarian stimulation for IVF and breast cancer risk in the studies conducted in the entire general population (relative risk (RR): 0.91, 95% CI: 0.74–1.11), nor in studies restricted to subfertile women (RR: 1.02, 95% CI: 0.88–1.18).

The role of oestrogens in the pathophysiology of breast cancer in the general population has only been partly elucidated. The dissimilarities in risks observed associated with exposure to long-lasting low levels of oestrogens, as is the case in oral contraceptives use and hormone replacement therapy,^4,5^ and with exposure to peak levels of oestrogens during a short period of time as in IVF are not yet understood. The existence of an oestrogen-receptor alpha (ERα)-dependent and -independent pathway has been suggested in breast carcinogenesis.^23^ The oestrogen-receptor-independent route consists of the enzymatic conversion of oestrogens into metabolites that damage the DNA.^24^ Since the *BRCA1* and *BRCA2* gene are involved in DNA repair, it is possible that the potential detrimental effect of oestrogens on mammary tissue is aggravated in *BRCA1/2* mutation carriers. This hypothesis is supported by the observation that hormonal factors have an influence on breast cancer risk in *BRCA1* mutation carriers, although effect directions are not consistent with observations in the general population.^25^ However, no associations were found between hormonal factors and breast cancer risk in *BRCA2* mutation carriers and in contrast to retrospective studies, a more recent study does not show a breast cancer risk reduction after RRSO in *BRCA1/2* mutation carriers.^22^ Prospective studies are needed to provide more insight into the aetiology of breast cancer in *BRCA1/2* mutation carriers and to further asses the involvement of hormonal factors in the pathophysiology.

There are several potential explanations for the lack of an effect of exposure to ovarian stimulation for IVF and the risk of breast cancer in our study: (1) there is an effect, but the effect size was too small to be detected due to limited power or observation time in the current study, (2) there is no true effect, as (a) ovarian stimulation for IVF has no influence on breast tissue carcinogenesis in *BRCA1/2* mutation carriers or (b) a protective influence of temporary low levels of oestrogens and progesterone due to downregulation of the natural cycle in IVF practice, which directs the effect size to the null, as suggested by van den Belt-Dusebout.^17^

In total, 3% of *BRCA1/2* mutation carriers included in the current study were exposed to IVF. The exposed group consisted of both women undergoing IVF because of subfertility as well as women opting for IVF because of PGD. After correction for oversampling by excluding the women ascertained via the national PGD registry, 2% of the women were exposed to IVF. In the general population, approximately 1% of all women are exposed to IVF.^26^ Several studies have suggested a reduced ovarian reserve in female carriers of a *BRCA1* mutation,^27–31^ but there is no convincing evidence for a clinically relevant adverse effect on fecundity.

Our study has several limitations. First, despite the availability of a nationwide *BRCA1* and *BRCA2* mutation cohort, questions regarding fertility treatments were only recently added to the questionnaire and power was still limited due to the low proportion of women exposed to IVF. To increase the power we included exposed carriers that were so far not participating in HEBON or were exposed to IVF after completion of the HEBON questionnaire, through a merge of the HEBON cohort with *BRCA1/2* mutation carriers included in the PGD registry. Although, in absolute numbers this was a very small increase, this addition increased the IVF-exposed group by 33%. There was a difference in data collection between the two sources, self-reported in a questionnaire versus medical records; however, it is very unlikely that IVF exposure (yes/no) was confounded by recall bias. The difference in age at censoring between the included groups HEBON and PGD is accounted for by the age-dependent analysis. Second, the subgroup of *BRCA2* mutation carriers was too small for a separate analysis (only three exposed *BRCA2* mutation carriers were diagnosed with breast cancer). Third, the retrospective study design may have resulted in survival bias if IVF-exposed women developed tumours with worse prognosis. Although the interval between breast cancer diagnosis and age at questionnaire was somewhat lower in IVF-exposed women compared to IVF-unexposed women (7.1 years ± 5.5 years versus 10.5 ± 8.0, respectively), this difference was not statistically significant (*p* = 0.052). Fourth, since data were self-reported-specific information on IVF protocols used was missing. In addition, in the questionnaire women were only asked to fill in their IVF history if they were subfertile. However, since fertile women with a PGD indication for IVF treatment were included by the PGD registry, only women opting for IVF treatment because of male subfertility have possibly been missed, which was the case in 22.3% of the population of IVF-exposed women.^17^ Lastly, in our retrospective cohort approach, breast cancer patients were oversampled due to the non-random uptake of DNA test. To account for this, a weighted cohort approach is suggested by Antoniou et al.,^32^ in which women with breast cancer and unaffected women are differentially weighted such that the breast cancer incidence rates in the study cohort are consistent with age- and birth cohort–specific breast cancer risk estimates for *BRCA1* and *BRCA2* mutation carriers. Unfortunately, power per age group was too limited to calculate reliable weights.

## Conclusion and recommendations

We did not find an increased risk of breast cancer after ovarian stimulation for IVF in *BRCA1/2* mutation carriers. Obviously, ruling out pre-existing lesions before an IVF treatment is started remains important, but based on the present knowledge there is no reason to exclude women with a *BRCA1/2* mutation from IVF for fertility treatment, fertility preservation or PGD. Still, larger studies with a longer time since IVF treatment are needed to exclude a small increased breast cancer risk and to investigate the long-term breast cancer risk for *BRCA1/2* mutation carriers.
